# “Frankness—Honesty—Truth”

**Published:** 1863-02

**Authors:** J. D. Anderson

**Affiliations:** Sacramento, Cal.


### Correspondence.

### "FRANKNESS—HONESTY—TRUTH."

### A REVIEW.

BY J. D. ANDERSON, D. D. S.

A man may cry, church, church, at every word,
With no more piety than other people;
A daw's not reckoned a religious bird
Because it keeps a cawing from a steeple.—Hood.

A robe of seeming truth and trust
Hide crafty observation.—Old Song.

The above quotations very naturally occurred to me when I read, in the November number of the Register, the elaborate piece of composition on Frankness, Honesty and Truth. If it is proper to deflect a Dental periodical from its ostensible purposes so far as to discuss, in its pages, the fundamental principles that beautify personal character, with which every school boy is familiar, and to turn such periodical into a sewer and fill it *ad nauseum*, with all the matter that can be eliminated from a "brain that labors, big with verse or prose," particularly prose, to be conducted out in the vast ocean of literature (and I do not object to this for a little sea sickness is occasionally desirable) as a bold cruiser in that ocean I trust I may, with perfect propriety, seize upon the craft, and before I release her, cortiously demand payment for my exploit as soon as I am recognized. So now I enter upon the work. Frankness, honesty and truth are carefully implanted in the plastic mind of youth by every hand that cultivates it, and in all Christian countries these qualities are regarded as the "diamonds of gold" that glitter most in the character of man. Indeed in all ages and throughout the world such qualities have been appreciated, and he who exercised them has not only been entitled to, but has received, the admiration and homage of his countrymen. But the writer of the piece I am reviewing, one would think, has just been made sensible of their beauty and importance by the urgent manner in which he assures us how *profitable* they are. Indeed from his labored argument and the interest he manifests in his fellow men, he seems desirous to impress it upon us that he has received a divine afflatus, visited the consecrated ground of Mt. Olives or Mecca, and had revealed to him a new polity for the benefit of a degenerate people. "Truth," says our spick and span new philosopher, has never yet been received as a policy of action in business." What a revelation! In all my travels, (and I have traveled some), I have never known an advocate of any other policy; and he who does not receive that policy and profess, at least, to adopt it, would be reprobated by an indignant community until very shame would drive him to the highway that he might escape the writhing scorn of every honest man. We might have excused our philosopher if he had confined himself to making out charges of corruption, falsehood, and duplicity against the world at large, for we should have regarded this as the sickly spleen of a misanthropic, moonstruck, madman; but not only do his exhortations imply that the dental profession is composed of dishonest and truthless men, but even has the assurance to announce that we hold our integrity so cheap as to invite our patients to gainsay us! Now, if he had been speaking to quacks who are constantly bringing our profession into disrepute, all this would have been well enough—would have been consistent with the last of the trinity of virtues that constitutes his text.

Such men, however, (*i. e.* quacks) as a body, never see a Dental journal; while that venerable class of men who have achieved an undying celebrity in building up a profession which they still adorn, together with that other and younger class who are characterized by a zeal and honesty of purpose which bid fair to emulate their examples, scrutinize every thing that appears in said journals; *ergo*, our philosopher was addressing himself to the elite, the respectability and the intelligent of a profession which seems to have honored him with the title of D. D. S., which he enjoys and parades on all occasions. Why he has been so ungrateful as to traduce the profession that has thus honored him, I can only attribute to that peculiar affection which surely troubles him—*cac-o-e-thes scribendi*—for in all my association with the dental profession I have never seen such evidences of dishonesty as would justify the exhortations our philosopher has favored us with. Truly I fear our philosopher is of that sect, one of whom thanked God he was not as other men, for while he "exhorts the brethren" to be truthful (which implies they are otherwise) he proclaims his own spotless integrity. But, oh, thou modern philosopher! thou who dost call up the names of Socrates and Franklin to attest the purity of thy doctrine, as a devotee at the shrines thou hast erected, as an humble admirer of the apothegms thy sapient pen has so cogently constructed, I implore thee to be seemingly consistent. For if in thy sage lectures thou sayest in the *naivete* of thy heart, that thou dost say to "a suspicious and close-fisted patient" that here is a small cavity and there a small cavity which need not now be filled, in order, as thou sayest, that "I may fill sundry other cavities, *at my own price*," suspicious and close observers may misconstrue thy meaning and call in question thy vaunted honesty.

Sacramento, Cal., *January* 5, 1863.

Dr. Morton, the alledged discoverer of the application of ether to practice, has again appealed to Congress for Compensation for the use of this agent in the Army. It will be remembered that a similiar claim was pressed through both houses several years ago, but failed to obtain the President's signature. Dr. Morton subsequently appealed to the medical profession for relief, and large sums of money were freely subscribed in Boston and New York towards a national testimonial. Recently the patent expired, and a renewal was refused. Dr. M. then instituted a suit against the N. Y. Eye Infirmary, but failed to establish the right of prosecution. He now returns to Congress, and renews his petition for compensation. If he was ever entitled to anything the Government should be the donor.—*Medical Times.*

